# Impaired heat shock protein 72 expression in women with polycystic ovary syndrome following a supervised exercise programme

**DOI:** 10.1007/s12192-019-01048-1

**Published:** 2019-11-16

**Authors:** Rebecca V Vince, Richard J Kirk, Myint M Aye, Stephen L Atkin, Leigh A Madden

**Affiliations:** 1 Sport, Health and Exercise Science, Faculty of Health Sciences, University of Hull, Hull, HU6 7RX UK; 2 Hull York Medical School, Faculty of Health Sciences, University of Hull, Hull, UK; 3 Weill Cornell Medical College, Ar-Rayyan, Qatar; 4 Biomedical Science, Faculty of Health Sciences, University of Hull, Hull, UK

**Keywords:** Hsp72, Polycystic ovarian syndrome, Heat shock protein

## Abstract

Induction of heat shock protein expression and the heat shock (stress) response are seen in exercise. This exercise-induced response is thought protective against cellular stress through the expression of heat shock proteins. The highly inducible heat shock protein 72 (HSP72) has been shown to be expressed in a number of stress-related conditions, but not investigated in women with polycystic ovary syndrome (PCOS). Twenty-one women (10 controls, 11 with PCOS) concluded an 8-week supervised, moderate-intensity exercise programme. Monocytes and lymphocytes were analysed by flow cytometry for HSP72 expression from blood samples prior to, mid-way and at the completion of the programme. The monocyte HSP72 expression showed an increase from baseline values through mid-way (*p* = 0.025), and at the completion of the programme (*p* = 0.011) only in the control group, the PCOS group showed no significant change. This pattern was similar for lymphocyte HSP72 expression where a significant increase was found at the completion of the programme (*p* = 0.01) only in the control group. The magnitude of increased HSP72 expression following completion of the programme was linked to baseline values only in the control group. In conclusion, increased HSP72 expression to exercise over an 8-week period was seen in control but not in PCOS women, suggesting that there is an impairment of HSP72 expression in response to exercise in these women.

## Introduction

Polycystic ovary syndrome (PCOS) is the most prevalent endocrine disorder affecting women of reproductive age (Azziz et al. 2004), and the prevalence in western populations estimated to be between 6 and 10% depending upon the diagnostic criteria used (Bozdag et al. 2016; Legro et al. 2013). The condition is characterised by disruption of the menstrual cycle with oligo- or amenorrhoea, hyperandrogenism often presenting with acne, seborrhoea and/or hirsutism, and polycystic ovaries (Chang et al. 2004; Ehrmann 2005; Moran and Teede 2009). PCOS is accompanied by a higher incidence of hypertension, insulin resistance (IR) and lipid profile abnormalities, resulting in an adverse cardiovascular profile (DeUgarte et al. 2005; March et al. 2010; Moran and Teede 2009; Sathyapalan and Atkin 2012; Toulis et al. 2011).

Heat shock proteins (HSPs) are present in most cells, including endothelial cells, and play an important role in normal cellular homeostasis and cell protection from damage in response to stress stimuli (Xu 2002). They are the largest family of transcriptionally regulated chaperone proteins that respond to cellular stress to help repair protein damage, preventing protein aggregation and degrading damaged proteins (Hooper 2007; Soti et al. 2005). The highly inducible 72-kDa heat shock protein (HSP72) has been researched in depth in both normal and stress-related conditions (Chung et al. 2008; Kregel 2002; Peart et al. 2011; Taylor et al. 2010). Primary roles include protection against stress-induced apoptosis (Mosser et al. 1997), and acting as a chaperone facilitating the folding of newly synthesised proteins, as well as the degradation of unstable proteins (Bukau and Horwich 1998). HSP72 expression has been shown to increase under a variety of conditions, such as exercise (Fehrenbach et al. 2005; Peart et al. 2011), cellular acidosis (Liu and Steinacker 2001) and hypoxia (Patel et al. 1995). HSP72 may therefore have a key role in patients with medical conditions due to their protective nature and increasing the basal levels of HSP72 has previously been studied in order to confer cellular protection against subsequent bouts of stress in an exercise setting (Madden et al. 2008; McClung et al. 2008; Sandstrom et al. 2008).

It has previously been shown that HSP72 expression in skeletal muscle was low in type II diabetes and IR patients, whereby the expression is inversely correlated with IR (Bruce et al. 2003; Kurucz et al. 2002). McCarty (2006) suggested that individuals with a low expression of HSP72 may be at higher risk of IR and subsequent diabetes development (McCarty 2006), and Chung et al. (2008) demonstrated there to be a reduced skeletal muscle HSP72 protein expression in obesity (Chung et al. 2008). Furthermore, women with PCOS were found to have increased serum HSP72 which was positively correlated with IR, markers of oxidative stress and inflammation (Gao et al. 2013), although a study on rats found that HSP70 serum expression was lower than a control group but expression was higher in ovarian tissue (Wu et al. 2019). A further study showed higher HSP90 expression in ovarian tissues of women with PCOS (Li et al. 2016).

Exercise itself is a form of physiological stress that induces an increase in the circulating concentrations of various stress markers, such as noradrenaline (NA) and HSP72 (Fleshner et al. 2003; Martin-Cordero et al. 2011). HSP function at the cellular level is to protect against any stressful conditions. Subacute activation of HSP results in stress tolerance and cytoprotection against what would be a lethal exposure to stress-induced molecular damage (Kalmar and Greensmith 2009). The induction of HSP therefore may have broad therapeutic benefits in the treatment of various diseases. Importantly, it has been shown that those small chaperone peptides that are able to stabilise protein confirmation and facilitate the removal of mutant proteins can indeed protect against IR and type II diabetes (Chung et al. 2008). This data has therefore led to the hypothesis that HSP72 may combat IR. Chung et al. (2008) also showed that humans with obesity and IR are associated with a decreased expression of HSP72 in skeletal muscle. A cyclical model proposed by Hooper and Hooper (2009) suggested that obesity-related inflammation promotes IR which contributes to the observed reduction in HSP expression in type II diabetes and that increasing HSP expression could in turn reverse this process. The aim of this initial pilot study was to assess the heat shock expression in circulating monocytes and lymphocytes and to investigate the response through an 8-week exercise programme in women with PCOS and healthy controls.

## Subjects and methods

All of the participants recruited for this study were aged between 18 and 40 years of age and gave informed consent after ethical approval was obtained (LREC: 10/H1313/44). Premenopausal PCOS women (recruited from outpatients at the Hull and East Yorkshire Diabetes, Endocrinology and Metabolism Clinic) were eligible if they showed characteristics of PCOS as described by the Rotterdam consensus (Chang et al. 2004). All control women had regular menses and no evidence of clinical or biochemical hyperandrogenism. Any volunteer suffering from an existing medical condition that was defined as a contraindication to exercise or injury was excluded from the study. At each initial visit, participants were screened via a pre-exercise medical questionnaire to highlight any contraindications to the test protocol. Participants with a BMI between 18.5 and 40.0 kg/m^2^ were deemed eligible for the study. Inclusion and exclusion criteria are shown in Table 1.

**Table 1:** Inclusion and exclusion criteria

Condition	Inclusion	Exclusion
PCOS	Aged 18–40, premenopausal women A BMI between 18.5 and 40.0 kg m^−2^ PCOS (as defined by Rotterdam consensus, 2003)	Pregnancy/breastfeeding/attempting to conceive History of CV, renal, hepatic and thyroid disease History of diabetes mellitus History of physical disability to exercise History of allergy to insulin/soy oil/purified egg (intralipid) Currently on oral antidiabetic medicine improving insulin sensitivity (such as metmorfin, weight reduction medication, e.g. orlistat, sibutramine) Family history of sudden death Regular exercise three times a week for the last 3 months
Controls	Same as PCOS arm	Identical to PCOS arm with the exception of no history of PCOS

### Baseline assessment

A maximal exercise test was required to prescribe the correct exercise intensity for subsequent sessions. The test was performed on a Woodway ELG55 motorised treadmill (Woodway, Weil an rhein, Germany) set at 0.1 % gradient throughout the test, which began at a speed of 4.5 km h^−1^ for 3.5 min. Gas collection was made using an Oxycon Pro Metabolic System (Jaegger, Hoechberg, Germany) throughout. Participants were asked to wear a facemask that covered the mouth and nose, and a rotary flow sensor was placed in the mouthpiece for gas collection. The Oxycon Pro was calibrated using a 3 ml Hans Rudolph volume calibrating syringe (Hans Rudolph model 5530, Kansas, USA). Participants wore a polar heart rate monitor (Polar Electro, OY, Finland) to monitor their heart rate (HR) throughout the testing procedure, and a safety harness was worn for the test procedure in order to ensure full safety. Readings of HR and VO_2_ kg^−1^ were manually recorded each minute. Participants were instructed to provide maximal effort and persist with the test until they felt they could no longer continue with exercising, or they reached 85% of their age predicted maximum heart rate (using Karvonen’s principle of 220-age). The VO_2_ data was 30-s stationary time-averaged, and the highest 30-s average in the incremental phase was regarded as the VO_2max_ (Midgley et al. 2009). The 30-s stationary time average provides a good compromise between removing noise and maintaining the underlying trend in relatively rapidly changing VO_2_ data (Midgley et al. 2009).

### Supervised exercise programme

Following baseline assessment, participants attended 3 exercise sessions per week for 8 weeks. Each session was scheduled to last 1 h based on participants’ ability. The programme used a standard protocol on either a Woodway ELG55 motorised treadmill or a HP Cosmos Pulsar Treadmill. Participants performed all sessions on the treadmill as closely as possible to 60% VO_2max_. VO_2_ kg^−1^ was measured after the warm-up, which lasted for 5 min at 4.5 km h^−1^ and for a period of 10 min. The exercise intensity was altered by changing the treadmill speed if this value was not within ± 2.5% of the target oxygen uptake. During exercise session visits, fluid intake was permitted ad libitum.

Heart rate (HR) and rate of perceived exertion (RPE) (Foster et al. 2001) were monitored every 15 min, and if the subject could not continue, they were able to stop if necessary. Alternatively, reducing exercise intensity for a period of time to assist recovery was also allowed. Each session finished with a 5-min cool down at 4.5 km h^−1^, and participants completed the session when HR returned to within 120% of normal values. The study subjects were asked to continue as per their own choices with regard to calorie intake throughout the programme.

### Further assessments

Participants repeated the baseline assessment within 72 h at the mid-way point of the programme (4 weeks). This mid-point assessment was used to evaluate the prescribed exercise intensity for the reminder of the programme if the individuals’ fitness showed improvement. After completion of the programme, a final assessment was undertaken at a scheduled visit.

### Hsp72 analysis

**Fig. 1:**
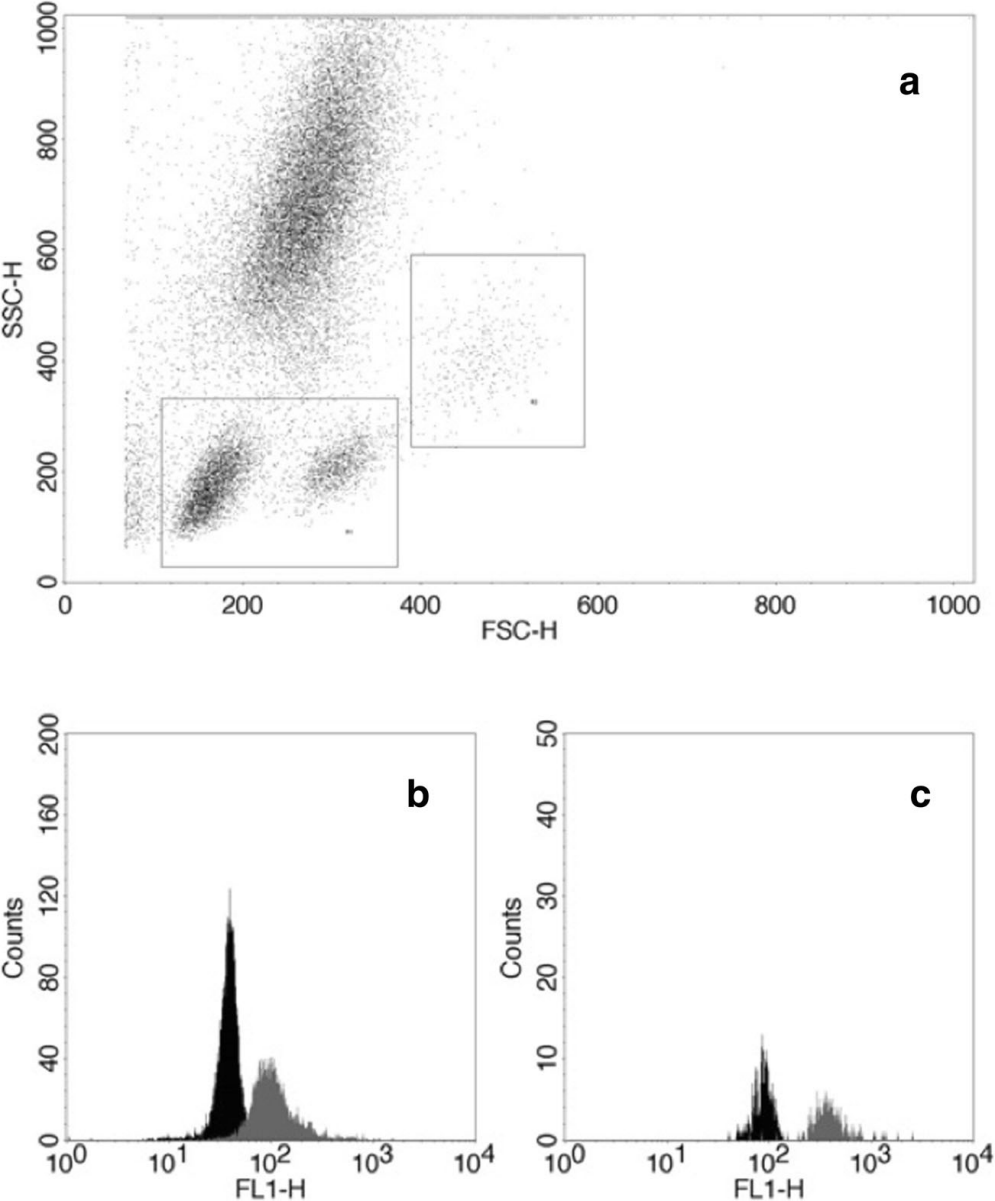
Typical gating strategy (**a**) and heat shock protein 72 expression (grey peak) in lymphocytes (**b**) and monocytes (**c**) compared to isotype-matched negative control (black peak) antibody labelling

The expression of intracellular HSP72 was measured by flow cytometry in both monocytes and lymphocytes using an established assay method (Sandstrom et al. 2009; Vince et al. 2010). Whole blood (100 μl) from EDTA tubes was transferred into a 2 ml red blood cell lysing buffer (Erythrolyse, AbDSerotec, UK) as a 1:10 dilution in distilled H_2_O for 10 min at room temperature. Samples were then centrifuged for 5 min at 300*g* in order to pellet white blood cells, and the resultant supernatant discarded. White blood cells were then washed with 2 ml PBS and centrifuged again at 300*g* for 5 min. Samples were then washed again with 2 ml PBS and centrifuged at 300*g* for 5 min then fixed with 100 μl of fix solution (Leucoperm Reagent A, AbDSerotec, UK) and left for 15 min at room temperature. Samples were then washed as above, and permeabilised by adding 100 μl of permeabilisation solution (Leucoperm Reagent B, AbDSerotec, UK). Each sample was then divided into two 50-μl aliquots. Then, 4 μl of either a negative control/FITC (AbDSerotec, UK) or IgG1 anti-HSP72/FITC antibody (Enzo Life Sciences, USA) was then added to these aliquots and left to incubate in the dark for 30 min. Samples were then washed with 2 ml PBS, centrifuged at 300*g* for 5 min and then resuspended in 300 μl PBS. Samples were analysed using flow cytometry on a BDFACS Calibur® (BD Biosciences, UK) running CELLQuest Software (BD Biosciences, UK). Monocytes and lymphocytes were gated by forward scatter (FSC; cell size) and side scatter (SSC; cell granularity properties), and a total of 20,000 events were counted (Fig 1a). Results were calculated as the ratio of the mean fluorescence intensity (MFI) gained with the anti-HSP antibody to that obtained with the isotype-matched negative control (Fig. 1b, c).

### Statistical analysis

Statistical analysis was performed using SPSS for Windows NT, version 19.0 (SPSS Inc., Chicago, IL). Changes in HSP72 (main effects) across condition (PCOS or control) and time (pre-, mid- and post-exercise programme) were analysed using linear mixed models. Post hoc tests with Sidak-adjusted *p* values were used to locate significant paired differences where a significant F ratio was observed. Subject characteristics were analysed between groups by independent *t* tests. Two-tailed statistical significance was accepted at *p* < 0.05.

## Results

### Subjects and baseline values

Twenty-five women were recruited onto the study, 10 out of 11 controls (91%), and 11 of 14 women (79%) with PCOS completed the programme and were included in the analysis. Baseline characteristics for control and PCOS women are shown in Table 2. There were significant differences in systolic blood pressure (SBP), VO_2max_, waist-hip ratio (WHR) and waist circumference (WC). There were no significant differences in either monocyte or lymphocyte HSP72 expression at baseline. BMI was higher in the PCOS group but did not quite reach statistical significance (*p* = 0.056). Clinical measures were repeated mid-point and after completion of the programme (Table 3) which showed a significant increase in VO_2max_ for both groups.

**Table 2:** Baseline characteristics between control and PCOS women. Age, body mass, blood pressure, VO_2max_, BMI, WHR, WC and HSP72 are compared at baseline between the two groups

Variables	Control baseline (*n* = 10)	PCOS baseline (*n* = 11)	*p* value
Age	24.26 ± 6.18	28.00 ± 6.72	0.202
Body mass (kg)	71.04 ± 16.42	85.45 ± 18.91	0.080
SBP (mmHg)	123.00 ± 11.44	132.36 ± 11.47	0.027*
DBP (mmHg)	77.50 ± 9.30	81.82 ± 11.21	0.290
VO_2_max (ml min^−1^ kg^−1^)	36.26 ± 6.38	26.32 ± 4.63	< 0.001*
BMI (kg m^−2^)	25.92 ± 5.39	31.15 ± 6.30	0.056
Waist-hip ratio	0.79 ± 0.07	0.86 ± 0.06	0.019*
WC (cm)	83.01 ± 14.20	98.05 ± 16.35	0.033*
Monocyte HSP72 (MFI)	5.10 ± 2.41	5.41 ± 2.14	0.756
Lymphocyte HSP72 (MFI)	3.51 ± 1.24	3.67 ± 1.29	0.308

*Significantly different between groups (*p* < 0.05). All data represented as mean ± SD

**Table 3:** Clinical characteristics of the control and PCOS women at baseline, mid and post 8 weeks of aerobic training

	Control (*n* = 10)			PCOS (*n* = 11)		
Variables	Pre	Mid	Post	Pre	Mid	Post
Body mass (kg)	71.04 ± 16.42	70.66 ± 16.19	70.11 ± 15.83	85.45 ± 18.91	84.45 ± 19.02*	84.04 ± 19.54*
Systolic blood pressure (mmHg)	123.00 ± 11.44	120.50 ± 10.28	117.50 ± 7.65*	132.36 ± 11.47	128.73 ± 11.03*	124.91 ± 10.40*†
Diastolic blood pressure (mmHg)	77.50 ± 9.30	75.00 ± 6.82	73.10 ± 5.43	81.82 ± 11.21	81.36 ± 12.36	76.64 ± 8.82*†
VO_2_ max (ml min^−1^ kg^−1^)	36.26 ± 6.38	37.49 ± 6.96	39.21 ± 5.82*	26.32 ± 4.63	26.43 ± 4.09	29.71 ± 5.32*†
BMI (kg m^−2^)	25.92 ± 5.39	25.78 ± 5.36	25.58 ± 5.18	31.15 ± 6.30	30.79 ± 6.32*	30.69 ± 6.48*
Waist to hip ratio	0.79 ± 0.07	0.78 ± 0.06	0.79 ± 0.07	0.86 ± 0.06	0.85 ± 0.07	0.85 ± 0.06
Waist circumference (cm)	83.01 ± 14.20	82.41 ± 14.19	82.16 ± 14.57	98.05 ± 16.35	96.97 ± 17.90	96.12 ± 14.67†

Data are represented as mean ± SD

*Significantly different compared to pre (*p* < 0.05)

†Significantly different compared to mid (*p* < 0.05)

### HSP72 expression through the exercise programme

**Fig. 2:**
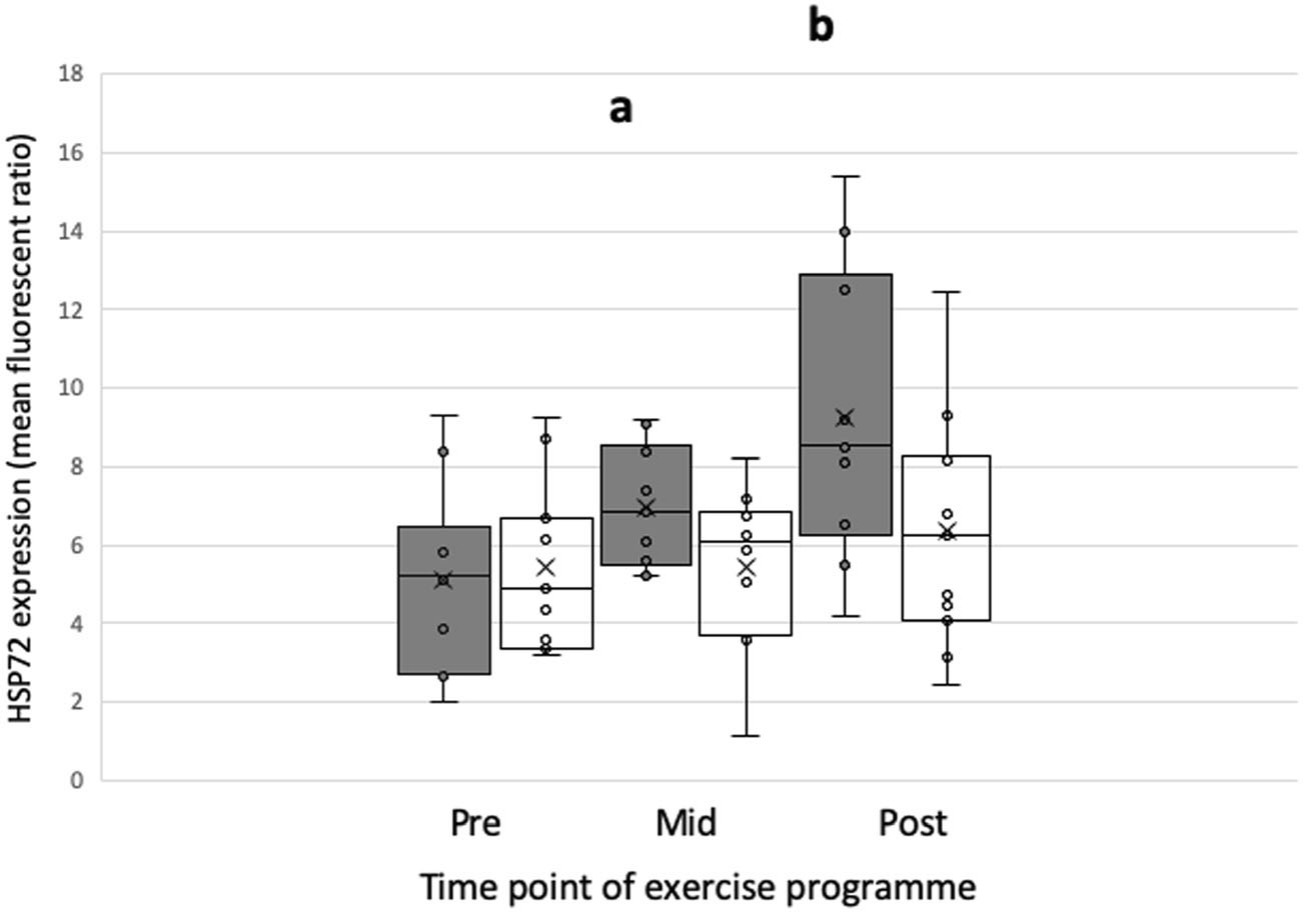
Levels of monocyte expressed HSP72 in controls (grey) and PCOS (white) participants at pre-, mid- and post-exercise intervention. ^a^Significantly different compared to pre in control group (*p* = 0.025). ^b^Significantly different compared to pre in control group (*p* = 0.011)

**Fig. 3:**
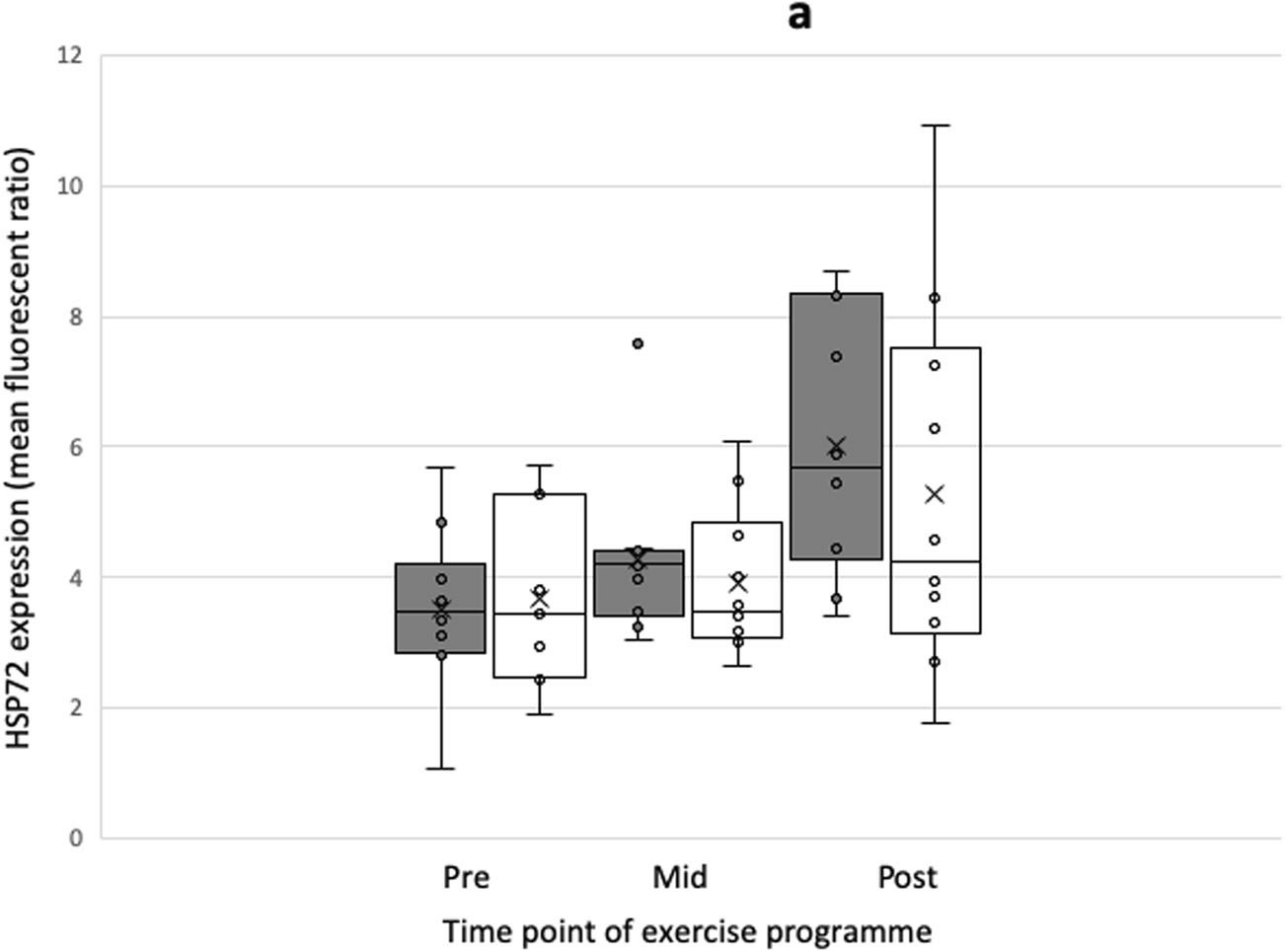
Levels of lymphocyte expressed HSP72 in controls (grey) and PCOS (white) in participants at pre-, mid- and post-exercise intervention. ^a^Significantly different to pre in control group (*p* = 0.01)

Flow cytometry gating of the target populations was based on well-established scatter properties (Fig. 1). The control population showed a significant increase (main effect for time) in monocyte-derived HSP72 from pre- to post-intervention, with a rise in MFI from 5.10 to 9.23, respectively (*p* = 0.011), and also pre- to mid-intervention, 5.10 to 6.98, respectively (*p* = 0.025, Fig. 2). The PCOS group also showed a rise from pre- to post-intervention (5.41 to 6.38, respectively), but this was not enough to see a significant change (*p* = 0.422). Lymphocyte HSP72 data followed a similar trend to the monocyte data as both groups showed a rise in HSP72 at each subsequent time point (Fig. 3). PCOS women saw a rise in MFI from 3.67 at rest to a peak of 5.27 post-exercise programme, although this was not significant (*p* = 0.155). The control women saw a significant rise in MFI from resting values at 3.51 to 6.03 at post-exercise programme (*p* = 0.01) but the rise from mid- to post-exercise was not significant (4.29 to 6.03, respectively, *p* = 0.083). The magnitude of the increase in HSP72 expression following the completion of the exercise programme was linked to baseline values in the control group in both monocytes (*p* = 0.030) and lymphocytes (*p* = 0.015), but this relationship was not observed in the PCOS group for either monocytes (*p* = 0.164) or lymphocytes (*p* = 0.212). No relationships were observed for HSP72 expression with BMI, body mass, age or VO_2max_ in either monocytes or lymphocytes at baseline, after completion of the programme, or overall.

## Discussion

The HSP72 response to an 8-week supervised, moderate-intensity exercise programme within circulating blood cells was shown to be impaired in women with PCOS compared to control women. The magnitude of increased HSP72 expression was non-significant in women with PCOS, whereas as may be expected, an increase over time was observed in controls. Various limitations exist in exercise interventions in women with PCOS (Harrison et al. 2011). They have generally been small in sample size and have had a significant drop out rate as also reported here. The ability to assess the possible influences of lean, overweight and obese individuals with and without PCOS was not possible due to the limited number of participants in each group. This study confirms that moderate intensity exercise (60% VO_2max_) could be recommended for this population.

Incorporating exercise as a treatment for PCOS may be favourable considering the benefits that exercise has in conditions that are associated with PCOS (Moran et al. 2006). Lifestyle modification has been endorsed by the Androgen Excess and PCOS Society as a primary choice in the prevention of CVD (Wild et al., 2010). There are well-established benefits of exercise and weight loss in women with PCOS on factors such as reproductive function (Palomba et al., 2008), and improvements in CVD risk markers such as obesity and cardiorespiratory fitness (Vigorito et al. 2007)*.* Other studies have also found improvements in women with PCOS as a result of moderate-intensity exercise (Palomba et al. 2010; Thomson et al. 2012), but future work could also investigate the use of incorporating high-intensity interval training or resistance training into a programme in PCOS women which has previously shown favourable results (Roessler et al. 2013).

The levels of obesity in both children and adults is increasing in developed countries and directly and indirectly impacts on healthcare budgets. Obesity is associated with IR, glucose intolerance and abnormal concentrations of lipids/lipoproteins which can lead to development of type 2 diabetes (Chung et al. 2008). PCOS is associated with low-grade inflammation and heat shock proteins have been studied previously in these patients, but not in response to an exercise programme. Studies have shown increased serum HSP, positively correlated with markers of oxidative stress, inflammation and IR (Gao et al. 2013). At baseline, in the present study, we observed no difference in monocyte or lymphocyte expression of HSP72 but did not investigate serum HSP72 levels.

Furthermore, obesity-related inflammation may be a factor in the impaired HSP72 expression observed here (Hooper & Hooper 2009), as the PCOS group had a higher body mass and BMI, but this was not statistically significant (Table 2). We showed that at baseline, no difference was observed in HSP72 expression within circulating blood cells between controls and women with PCOS. It has been proposed that a loss of the heat shock response disrupts metabolic homeostasis which can lead to a number of pathologies (Hooper et al. 2014). The beneficial effects of exercise are linked to the ability of cells to respond to stress and an impaired stress response has also been linked to pathogenesis of type 2 diabetes (Hooper et al. 2014). Previously, we have shown in vitro that HSP72 induction is dependent on both temperature and time (Lovell et al. 2007) and that HSP72 expression in monocytes is linked to core temperature in human subjects (Sandstrom et al. 2009). Furthermore, increasing the basal levels of HSP72 has previously been shown to confer cellular protection against subsequent bouts of stress in an exercise setting (Madden et al. 2008; McClung et al. 2008; Sandstrom et al. 2008) and the increase in HSP72 reported here was linked to baseline values in the control group, suggesting that higher baseline values are beneficial in adaptation to stress; however, the PCOS group did not have the same relationship suggesting an impairment of HSP72 expression. Both groups exercised at a standardised 60% of VO_2max_; however, the baseline VO_2max_ was significantly different between groups (Table 2), and therefore, the level of exercise output and intensity may be lower in the PCOS and not enough to elicit an HSP72 increase. VO_2max_ also significantly increased in both groups following completion of the programme (Table 3). A limitation of the study is a relatively low sample size of 25 in total, and the control population was not matched well in terms of body composition and VO_2max_, although there was no significant difference in HSP72 at baseline between the groups. Whilst heat shock expression within monocytes is an ideal choice for investigating the heat shock status (Bachelet et al. 1998) within the circulation, it only offers an insight based on this cell population.

Induction of heat shock proteins may not only act to improve IR (Hooper & Hooper 2009) and overall metabolic health, but has also been proposed to potentially alleviate microvascular and macrovascular complications associated with diabetes (McCarty 2006), although as highlighted here, although the PCOS did show an increase in HSP72 expression, this was not at the levels seen in the control group. To achieve higher HSP72 through exercise in women with PCOS may require a longer programme of exercise as increasing the intensity of the programme above 60% VO_2max_ would not be suitable for this population and would likely result in an increased drop-out rate.

## Conclusion

A supervised exercise programme devised was suitable for women with PCOS who displayed an impaired HSP72 expression when compared with controls.
